# Protease-Mediated Growth of Staphylococcus aureus on Host Proteins Is *opp3* Dependent

**DOI:** 10.1128/mBio.02553-18

**Published:** 2019-04-30

**Authors:** McKenzie K. Lehman, Austin S. Nuxoll, Kelsey J. Yamada, Tammy Kielian, Steven D. Carson, Paul D. Fey

**Affiliations:** a Department of Pathology and Microbiology, University of Nebraska Medical Center, Omaha, Nebraska, USA; b Department of Biology, University of Nebraska at Kearney, Kearney, Nebraska, USA; University of Minnesota Medical School

**Keywords:** *Staphylococcus aureus*, amino acid catabolism, metabolism, proteases

## Abstract

Staphylococcus aureus has the ability to cause infections in multiple organ systems, suggesting an ability to rapidly adapt to changing carbon and nitrogen sources. Although there is little information about the nutrients available at specific sites of infection, a mature skin abscess has been characterized as glucose depleted, indicating that peptides and free amino acids are an important source of nutrients for the bacteria. Our studies have found that mutations in enzymes necessary for growth on amino acids, including pyruvate carboxykinase (Δ*pckA*) and glutamate dehydrogenase (Δ*gudB*), reduced the ability of the bacteria to proliferate within a skin abscess, suggesting that peptides and free amino acids are important for S. aureus growth. Furthermore, we found that collagen, an abundant host protein that is present throughout a skin abscess, serves as a reservoir of peptides. To liberate peptides from the collagen, we identified that the host protease, MMP-9, as well as the staphylococcal proteases aureolysin and staphopain B function to cleave collagen into peptide fragments that can support S. aureus growth under nutrient-limited conditions. Moreover, the oligopeptide transporter Opp3 is the primary staphylococcal transporter responsible for peptide acquisition. Lastly, we observed that the presence of peptides (3-mer to 7-mer) induces the expression of aureolysin, suggesting that S. aureus has the ability to detect peptides in the environment.

## Abstract (precis)

Staphylococcus aureus has the ability to cause infections in a variety of niches, suggesting a robust metabolic capacity facilitating proliferation under various nutrient conditions. The mature skin abscess is glucose depleted, indicating that peptides and free amino acids are important sources of nutrients for S. aureus. Our studies have found that mutations in both pyruvate carboxykinase and glutamate dehydrogenase, enzymes that function in essential gluconeogenesis reactions when amino acids serve as the major carbon source, reduce bacterial burden in a murine skin abscess model. Moreover, peptides liberated from collagen by host protease MMP-9 as well as the staphylococcal protease aureolysin support S. aureus growth in an Opp3-dependent manner under nutrient-limited conditions. Additionally, the presence of peptides induces aureolysin expression. Overall, these studies define one pathway by which S. aureus senses a nutrient-limiting environment and induces factors that function to acquire and utilize carbon from host-derived sources.

## INTRODUCTION

Bacteria that colonize or infect a susceptible host must evade the immune response as well as scavenge appropriate host nutrients in order to proliferate. Significant advances have been made in understanding the regulation and function of factors that aid in the establishment of an infection and the evasion of the immune response (1, 2), but we are only beginning to understand the carbon and nitrogen metabolism required for bacteria to thrive within a particular niche.

Staphylococcus aureus is a predominant cause of skin and soft-tissue infections (3), which commonly present as primary pyoderma or abscesses. Abscesses begin as a localized host immune response that functions to limit the bacterial burden and subsequent tissue damage (4). Acute abscesses are characterized by live bacteria, neutrophil infiltrates, tissue debris, and fibrin (2). As the abscess matures, fibroblasts mediate tissue repair and form a fibrous capsule surrounding the abscess, composed of fibrin and type I collagen (5). Abscess formation is an active process that requires both a host immune response as well as staphylococcal virulence factors and surface proteins, including fibrinogen-binding proteins ClfA and ClfB, coagulase, von Willebrand factor-binding protein, heme-scavenging proteins IsdA and IsdB, SdrC, and protein A (2, 4, 6). In addition, dermonecrosis, which is often associated with an S. aureus skin abscess, is dependent upon the pore-forming toxin alpha hemolysin (*hla*) (7–10).

Although the nutrients available within particular niches of the host are not well characterized, staphylococcal glycolytic activity is required to initiate an infection in mouse models of disease (11, 12). The dependency upon glycolysis is due, in part, to the ability of S. aureus to ferment glucose and generate ATP using a nitric oxide (NO·)-insensitive lactate dehydrogenase to facilitate redox balance (12–14). Activated phagocytes utilize inducible nitric oxide synthase (iNOS) to generate NO· (15), which inhibits bacterial replication and respiration (16, 17). Therefore, in contrast to other staphylococcal species, S. aureus is highly resistant to this antimicrobial free radical, largely due to its expanded metabolic repertoire that allows the bacteria to circumvent the NO· damaged respiratory chain (12, 14).

As noted, staphylococcal glycolysis is required for skin abscess formation in a murine model, suggesting that resistance to NO· stress is essential to this process. However, once the abscess is formed, the bacteria replicate within the abscess in what has been called the staphylococcal abscess community (2). This environment is thought to be hypoxic, limiting the production of NO· and relieving the requirement for glycolysis (12, 18). Moreover, the low-oxygen environment induces HIF-1α expression within the infiltrating host immune cells, driving glucose consumption (11, 19). Recent studies have confirmed that as a skin abscess matures, there is a reduction in oxygen and glucose levels (20). In this glucose-depleted environment, S. aureus proliferation likely requires the use of alternative carbon sources, such as the lactate excreted by S. aureus and peptides from the extracellular milieu. Lactate is utilized via lactate quinone oxidoreductase (Lqo), which generates pyruvate and, subsequently, acetate and ATP via Pta/AckA, whereas peptides provide a carbon source for gluconeogenic reactions. Indeed, work by our laboratory has found that S. aureus utilizes glutamate and those amino acids that can be used as substrates to synthesize glutamate (proline, arginine, and histidine) as primary carbon sources in media lacking glucose (21). Lastly, growth on amino acids is preferred in manganese-depleted environments such as an abscess due to the dependency on this metal for glycolytic enzymes (22).

S. aureus produces a variety of secreted proteases that not only serve as virulence factors by cleaving staphylococcal surface proteins (23–25), degradation of host tissue (26, 27), and modulation of the host immune response (28–30) but also aid in nutrient acquisition from the host (31). The proteases encoded by S. aureus include two cysteine proteases, staphopain A (ScpA) and staphopain B (SspB), a metalloproteinase, aureolysin (Aur), a serine protease (V8 or SspA), and serine protease-like proteins (Spls). The operons that encode these proteases are positively regulated by the quorum-sensing system, Agr, and negatively regulated by SarA (32, 33). While the Spl proteins are secreted as active proteases, Aur, ScpA, V8, and SspB require proteolytic activation. Aur and ScpA autoactivate extracellularly (34, 35). Once active, Aur activates V8 protease (36), and V8 then cleaves and activates SspB (37). When grown in peptide-rich media or in serum, a protease-null strain of S. aureus has reduced fitness compared to that of wild-type strains, suggesting that secreted proteases are important for nutrient acquisition (31). Additionally, proteases have been shown to cleave specific host proteins. For instance, SspB can degrade collagen, human fibronectin, and fibrinogen (26, 37, 38). In addition to cleaving staphylococcal surface proteins, including fibronectin binding protein, protein A, and clumping factor B (ClfB) (24, 25), Aur also cleaves host proteins. Host targets of Aur include the complement factor C3, antimicrobial peptide LL-37, and plasminogen (39–41). *In vivo*, secreted proteases appear to be important in a skin and soft-tissue infection, as a protease-null strain has significantly reduced bacterial burden in this model (31).

Nutrient acquisition in the host requires transport systems for uptake of nutrients that are required for proliferation. When grown in medium containing free amino acids and peptides as major carbon sources, S. aureus preferentially consumes free amino acids, and, once free amino acids are depleted, peptides are consumed (42). Opp import systems are found in Gram-positive and -negative bacteria and belong to a large family of ATP-binding cassette (ABC) transporters that hydrolyze ATP to drive transport (43). The typical Opp transport system is comprised of five proteins, including the oligopeptide binding protein, OppA, the transmembrane proteins, OppB and OppC, which form the channel for peptide translocation, and two membrane-bound cytoplasmic ATP-binding proteins, OppD and OppF (44). S. aureus JE2 encodes six putative peptide transport systems, including five complete oligopeptide permeases (Opp1ABCDF, Opp2BCDF-Opp5A, Opp3BCDFA, Opp4ADFBC, and OppACME-ABCDF) and one dipeptide permease (DtpT) (42, 45). Only one of the Opp transport systems, Opp3, along with the dipeptide transporter, DtpT, have been shown to be important for peptide acquisition in S. aureus (45). Two of the Opp loci, Opp2BCDF and Opp5A, have been identified to work together as a nickel transporter (renamed NikBCDF and NikA, respectively) (46, 47), and Opp1ABCDF has been identified as a cobalt and nickel transporter (renamed CntABCDF) (48). Notably, Opp3 is the only Opp system that is conserved in all staphylococcal species (49).

The ability of S. aureus to proliferate in a variety of niches within the host suggests that the bacterium has a flexible metabolism that allows for growth in the presence or absence of key carbon sources, such as glucose. The current study defines the skin abscess as a niche in the host that is likely glucose depleted and therefore requires S. aureus to utilize peptides and free amino acids as its primary carbon source. Moreover, collagen, a protein that is relatively abundant in a skin abscess, can be utilized by S. aureus as a source of essential nutrients. Collagen is degraded into peptide fragments by the host protease MMP-9 as well as staphylococcal proteases. The peptide fragments are transported into S. aureus by the oligopeptide transporter Opp3, where the peptide can be catabolized as a carbon and nutrient source. Additionally, the presence of peptides induces the expression of S. aureus proteases, suggesting that the bacterium has a mechanism of sensing environments in which peptides are available for proliferation. Overall, these studies highlight the ability of S. aureus to rapidly adapt to its host environment and proliferate within a specific host niche.

## RESULTS

### S. aureus skin abscess persistence requires gluconeogenesis and glutamate catabolism.

**FIG 1:**
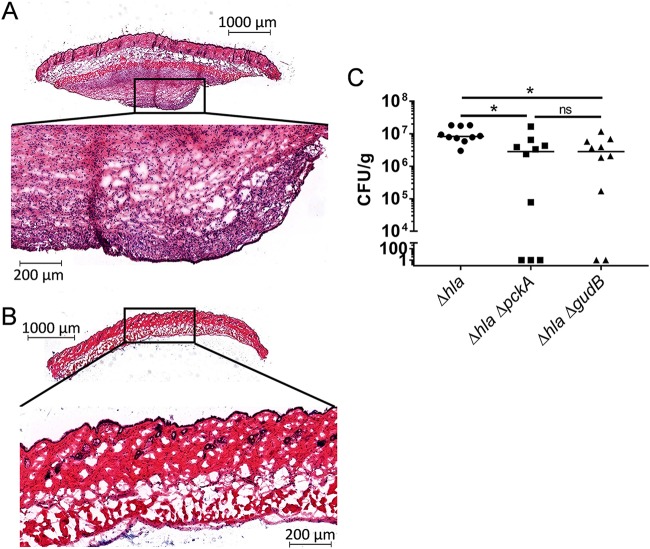
Gluconeogenesis and amino acid catabolism are important for cellular proliferation in an S. aureus skin abscess. (A) Magnification (2×) of hematoxylin and eosin staining of 5-day skin abscess and surrounding periabscess tissue from 7-week-old C57BL/6 mice infected subcutaneously with 1 × 10^6^ CFU S. aureus Δ*hla* strain, resulting in abscess formation. The inset represents the abscess magnified 10×, showing the infiltration of immune cells to the area of the infection. (B) Magnification (2×) of hematoxylin and eosin staining of control tissue from 7-week-old C57BL/6 mice. The inset represents the area magnified 10×, showing a lack of immune cell infiltrate. (C) Bacterial burdens of 7-week-old C57BL/6 mice subcutaneously infected with 1 × 10^6^ CFU of S. aureus Δ*hla*, Δ*hla* Δ*pckA*, or Δ*hla* Δ*gudB* strain 5 days postinfection. Statistical significance was determined by Kruskal-Wallis test. *, *P* < 0.05, *n* = 10. ns, not significant.

Although glucose is a primary carbon source for S. aureus, there are likely niches in the host where glucose is not available and other carbon sources must be utilized. When glucose is not available, S. aureus has the metabolic capability to catabolize lactate, acetate, and amino acids (14). Moreover, glutamate and the amino acids that can be converted into glutamate (proline, arginine, and histidine) provide the majority of the carbon required for cell proliferation (21). We sought to determine if a mature skin abscess is dependent upon gluconeogenesis and glutamate catabolism, indicating that glucose is indeed limited and alternative carbon sources such as peptides and amino acids are required. It is important to note that while utilizing the soft-tissue infection model as previously described (5), we observed two distinct disease phenotypes in the animals. The first was a walled-off abscess with little to no associated dermonecrosis (see Fig. S1A in the supplemental material). The second was a dermonecrotic lesion in which there was clear necrosis of the dermis and epidermis and an underlying abscess (Fig. S1B). It was evident that these two disease presentations resulted in different environments for the bacteria to proliferate and, therefore, represented distinct niches. To study a more homogeneous disease outcome, we utilized an *hla* mutant (Δ*hla*) that resulted solely in skin abscess formation (7), with little to no dermonecrosis associated with the infection (Fig. 1A).

10.1128/mBio.02553-18.1 FIG S1 Images of 7-week-old C57BL/6 mice infected with 1 × 10^6^ CFU S. aureus JE2, resulting in skin abscess formation (A) or dermonecrotic lesion (B). (Left) Image of mouse prior to abscess or necrotic lesion excision. (Right) Underside of the abscess or necrotic lesion prior to excision. The red dashed line outlines each lesion. Download FIG S1, PDF file, 0.2 MB. Copyright © 2019 Lehman et al. 2019 Lehman et al. This content is distributed under the terms of the Creative Commons Attribution 4.0 International license.

To determine if gluconeogenesis is required for survival and proliferation within a skin abscess, a gluconeogenesis mutation, Δ*pckA*, was introduced into the Δ*hla* background. In a 5-day skin abscess infection, we observed bacterial levels below the level of detection in 30% of the animals infected with the Δ*hla* Δ*pckA* strain (Fig. 1C), suggesting that gluconeogenesis and, thus, growth on secondary carbon sources is important for survival and proliferation within a skin abscess. Furthermore, we introduced a glutamate dehydrogenase mutation (Δ*gudB*) into the Δ*hla* background. In previous studies, *gudB* was found to be essential for growth in media where amino acids were the sole carbon source, since glutamate, proline, arginine, and histidine cannot be utilized as carbon sources in this mutant (21). Similar to what we observed in the *Δhla ΔpckA* mutant, 20% of mice infected with the Δ*hla* Δ*gudB* mutant had bacterial burdens below the level of detection after 5 days (Fig. 1C). Together, these data suggest that as the abscess matures, glucose is limited, forcing the bacteria to rely on amino acid catabolism and gluconeogenesis to proliferate within the abscess.

### Host protease MMP-9 is upregulated at the site of S. aureus infections.

Our data suggest that amino acid catabolism is important for S. aureus growth in murine skin and soft-tissue abscesses. As glutamate is the major carbon source when S. aureus is growing in glucose-limited environments, we hypothesize that acquisition of peptides containing glutamate, or those amino acids that can be used as substrates to synthesize glutamate, such as proline, arginine, and histidine, are important for S. aureus proliferation within a skin abscess. We further propose that acquisition of proline or arginine is fundamentally important, as they can serve as precursors for proline, arginine, and glutamate synthesis under glucose-depleted conditions, whereas glutamate and histidine are not substrates for synthesis of proline and arginine. However, several lines of evidence suggest that the milieu within an inflammatory response is arginine depleted due to host iNOS and arginase-1 expression, further highlighting the potential importance of proline as a substrate for arginine and/or glutamate synthesis (5, 50, 51).

**FIG 2:**
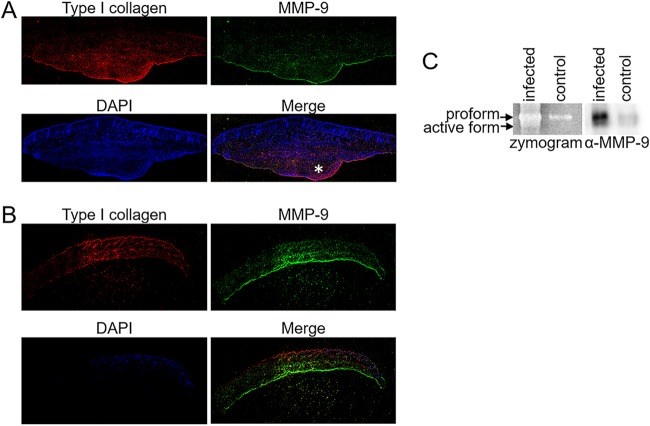
Host protease MMP-9 is upregulated in infected tissue. (A and B) Confocal micrographs of the skin abscess and surrounding periabscess tissue (A) or control tissue (B) from 7-week-old C57BL/6 mice subcutaneously infected with 1 × 10^6^ CFU S. aureus Δ*hla* strain after 5 days. Tile-scanned images were taken of sections stained with MMP-9 (green), type I collagen (red), and 4′,6-diamidino-2-phenylindole (DAPI) (blue). An asterisk denotes the location of the abscess. (C) Two μg of protein from homogenized control and S. aureus Δ*hla* strain-infected tissues was run either on a 10% SDS-PAGE gel embedded with 0.01% gelatin (zymogram) or 10% SDS-PAGE gel (Western blot). MMP-9 is detected in both its zymogen and activated form, as indicated by the arrows.

Collagen is an abundant host protein and is found within the fibrotic wall and tissue surrounding the abscess (Fig. 2A). Furthermore, proline is one of the most abundant amino acids in collagen. The host produces a variety of proteases that are able to degrade collagen, including MMP-1, -2, -8, -9, and -13 (52). MMP-9 functions in the remodeling of the extracellular matrix and has been shown to be important in neutrophil extravasation, wound healing, and bone remodeling (53). In earlier work, we studied the importance of arginine biosynthesis in kidney abscess proliferation (5). To follow up on this work and determine host factors that are induced during abscess formation, kidney homogenate from S. aureus JE2- and mock-infected mice were separated on an SDS-PAGE gel. A unique band from the infected kidney homogenate was analyzed by liquid chromatography-tandem mass spectrometry (LC-MS/MS) and identified as MMP-9. These data were confirmed using a collagen zymogram, documenting that collagenase activity was dependent upon the presence of calcium, and MMP-9 enzymatic activity is known to be calcium dependent (54) (Fig. S2A). Moreover, kidney homogenate from S. aureus JE2-infected MMP-9 knockout mice no longer displayed collagenase activity in the zymogram (Fig. S2B). We hypothesized that the host protease MMP-9 is important for liberation of nutrients from host proteins that allow for S. aureus proliferation within a skin abscess. We confirmed that MMP-9 is also abundant in a skin abscess, and, similar to findings for a kidney abscess, MMP-9 is more abundant in infected tissue than in control tissue (Fig. 2C).

10.1128/mBio.02553-18.2 FIG S2 (A) Supernatant from uninfected kidney and S. aureus JE2-infected kidney homogenate was run on a 10% SDS-PAGE gel with 0.5 mg ml^−1^ type I collagen added. On the left, a 100-kDa collagenase (black arrow) was present in the infected kidney homogenate lane but absent from the uninfected kidney homogenate lane. The collagenase activity was abolished when EDTA was added. (B) Collagen zymogram of S. aureus JE2-infected kidney homogenate from MMP-9 knockout and C57BL/6 mice. Download FIG S2, PDF file, 0.2 MB. Copyright © 2019 Lehman et al. 2019 Lehman et al. This content is distributed under the terms of the Creative Commons Attribution 4.0 International license.

### S. aureus proteases are important for MMP-9 activation in addition to collagen degradation.

**FIG 3:**
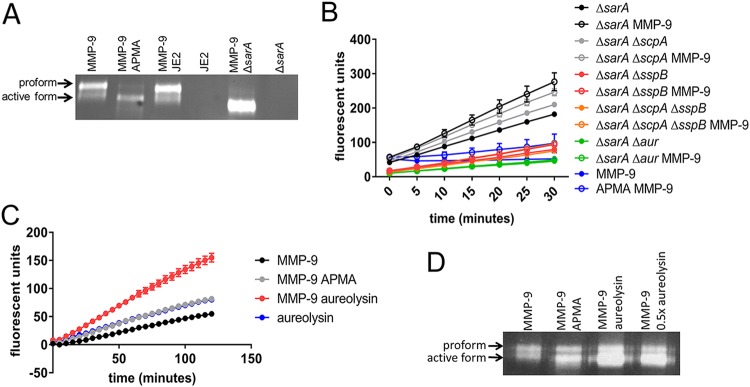
Aureolysin activates MMP-9 and cleaves collagen. (A) Zymogram of purified MMP-9 incubated with the following: 1 μM APMA, S. aureus JE2 supernatant, or S. aureus JE2 Δ*sarA* strain supernatant. Two ng of MMP-9 was run on a 10% SDS-PAGE gel embedded with 0.01% gelatin. After renaturation, the gel was counterstained with Coomassie dye to reveal the zones of clearing. Both the proform and active form can be identified as indicated by the arrows. (B) MMP-9 (10 ng) and various S. aureus JE2 Δ*sarA* mutant strain supernatants were incubated with 12.5 μg DQ collagen. Cleavage of DQ collagen resulted in increased fluorescence. Data are represented as means ± standard errors of the means (SEM; *n* = 3). (C) Purified aureolysin (10 ng) and/or pro-MMP-9 (10 ng) were incubated with DQ collagen to assess collagen cleavage. One μM APMA was used as a chemical activator of MMP-9. Data are represented as means ± SEM (*n* = 3). (D) Zymogram of purified MMP-9 (2 ng) incubated with 1 μM APMA or aureolysin (2 ng or 1 ng).

We observed both the proform and active form of MMP-9 in the infected tissue (Fig. 2C), demonstrating that MMP-9 not only is present during an S. aureus infection but also is activated. Previous studies have demonstrated that bacterial proteases have the ability to activate various MMPs, including MMP-9 (55–58). To first determine if S. aureus secretes a protease capable of activating MMP-9, a gelatin zymogram was performed. In this assay, purified human MMP-9 was incubated with the overnight (18 h) supernatant of wild-type S. aureus JE2 or the *sarA*::ΦΝΕ transposon mutant (Δ*sarA*) grown in the rich medium Trypticase soy broth (TSB). The Δ*sarA* strain is known to overexpress all S. aureus proteases (32, 59, 60). As shown in Fig. 3A, incubation of MMP-9 with APMA, a chemical activator of MMP-9, resulted in activation of the protease. Additionally, incubation with Δ*sarA* supernatant, but not wild-type supernatant, resulted in MMP-9 activation. These data indicate that one of the proteases overexpressed in the *sarA* mutant has the ability to activate MMP-9. To elucidate the responsible secreted protease(s), we transduced the *sarA* mutation into the Δ*aur*, Δ*scpA*, Δ*sspB*, and Δ*scpA* Δ*sspB* mutant backgrounds and observed the cleavage of fluorescently labeled collagen (DQ collagen) by spent medium in the presence and absence of purified pro-MMP-9. Supernatants from Δ*sarA* mutant containing pro-MMP-9 had the highest rate of collagen cleavage (Fig. 3B), nearly five times the rate of APMA-activated MMP-9 (Table S1). Moreover, Δ*sarA* supernatant alone produced elevated levels of collagen cleavage, suggesting that a secreted S. aureus protease also has collagenase activity. When the *aur* mutation was introduced into the Δ*sarA* background, there was a large reduction in collagenase activity in the supernatant, suggesting Aur, or the staphylococcal proteases it activates, has the ability to cleave collagen. Previous studies have demonstrated that ScpA and SspB also have collagenase activity (26). We observed that the supernatant from the Δ*sarA* Δ*sspB* mutant had reduced collagenase activity in the presence and absence of pro-MMP-9, although it was not as dramatic as the effect of an *aur* mutation, suggesting that SspB contributes to the degradation of collagen when overexpressed in this assay. Importantly, the rates of collagen cleavage by the supernatant from the Δ*sarA* Δ*sspB* mutant was significantly (*P* = 0.0001) reduced compared to that of Δ*sarA* Δ*sspB* mutant MMP-9, suggesting that MMP-9 is active and cleaving collagen in the absence of SspB. Supernatant from the Δ*scpA* Δ*sarA* mutant cleaved collagen at the same rate as the *sarA* mutant (*P = *0.1386), indicating that ScpA does not have the ability to cleave collagen under the conditions tested. Supernatant from the Δ*scpA* Δ*sspB* Δ*sarA* mutant phenocopied the collagenase activity of the Δ*sspB* Δ*sarA* mutant, further documenting the function of SspB in the proteolysis of collagen.

10.1128/mBio.02553-18.5 TABLE S1 Rates of collagen degradation. Download Table S1, DOCX file, 0.01 MB. Copyright © 2019 Lehman et al. 2019 Lehman et al. This content is distributed under the terms of the Creative Commons Attribution 4.0 International license.

Aur is the first secreted protease in the proteolytic cascade that activates V8 and SspB. We therefore sought to determine if the inability to cleave collagen in the Δ*sarA* Δ*aur* double mutant was because of the inability to activate downstream proteases or if Aur functions to cleave collagen. In this assay, purified Aur was used in the presence or absence of pro-MMP-9. Purified Aur had the ability to cleave collagen at a rate similar to that of chemically activated MMP-9 (MMP-9 APMA) (Fig. 3C). Moreover, the addition of pro-MMP-9 enhanced the rate of collagen cleavage, demonstrating that Aur is the S. aureus protease responsible for the activation of MMP-9 (Fig. 3A). Gelatin zymography confirmed that Aur has the ability to activate MMP-9 (Fig. 3D). Together, these data indicate that Aur and SspB both function to cleave collagen, and Aur has the ability to activate MMP-9, which also has collagenase activity.

### MMP-9- and aureolysin-digested collagen supports S. aureus growth under nutrient-limited conditions.

**FIG 4:**
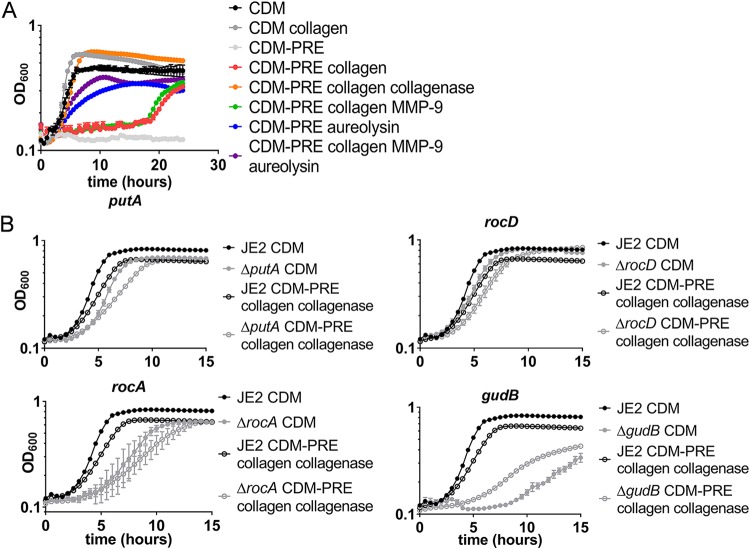
Digested collagen can support growth in media lacking essential amino acids. (A) Growth curves of S. aureus JE2 grown in CDM, CDM-PRE, or CDM-PRE supplemented with the following: collagen, collagen treated with collagenase, collagen treated with MMP-9, collagen treated with aureolysin, collagen treated with MMP-9, and aureolysin. Data are represented by the means ± SEM (*n* = 3). (B) Growth curves of S. aureus JE2, Δ*putA*, Δ*rocD*, Δ*rocA*, and *ΔgudB* strains in CDM and CDM-PRE supplemented with collagen treated with collagenase. Data are represented as means ± SEM (*n* = 3).

The previous data showed that the host protease MMP-9, as well as the staphylococcal proteases Aur and SspB, have the ability to degrade collagen, an abundant host protein that is present at the site of S. aureus skin infections (Fig. 2A). As peptides are likely an essential nutrient during growth in glucose-depleted media, we sought to determine if collagen can support growth in chemically defined medium (CDM) lacking glucose, proline, arginine, and glutamate (CDM-PRE) supplemented with collagen digested with various proteases. S. aureus is auxotrophic for either proline or arginine and requires an exogenous source of these amino acids during growth in CDM. Collagen treated with collagenase from Clostridium histolyticum was used as a positive control and could restore bacterial growth in CDM-PRE to levels seen in complete CDM (Fig. 4A). When collagen was digested with Aur, we observed a rapid restoration of growth, suggesting that aureolysin could digest the collagen into available peptides and free amino acids. Moreover, the combination of pro-MMP-9 and Aur also rapidly promoted growth under these conditions, only mildly enhancing the growth rate and yield over that of collagen treated with Aur, suggesting that both proteases cleave collagen into usable substrates for S. aureus peptide uptake. In contrast, S. aureus grown with collagen pretreated with pro-MMP-9 had a delayed recovery of growth, suggesting that the bacteria had to produce an additional factor, likely an endogenous protease, to activate MMP-9 and/or help digest the collagen to a form that could be easily consumed. Interestingly, there was also growth, albeit delayed, of S. aureus in the CDM-PRE with collagen, suggesting that over time, S. aureus produces enough protease to eventually degrade collagen into peptides that can support growth.

Additionally, we sought to determine whether catabolism of a particular amino acid is essential for growth on degraded collagen by using various mutations in the arginine, proline, and glutamate catabolic pathways. Again, CDM-PRE was supplemented with collagenase-digested collagen. It should be noted that all of the mutants tested have a modest (Δ*putA*, Δ*rocA*, and Δ*rocD* mutants) or severe (Δ*gudB* mutant) growth defect in CDM (21), as these enzymes function in pathways that catabolize the primary carbon sources in this medium. All of the mutants grew on CDM-PRE supplemented with collagenase-treated collagen at rates similar to those of the respective mutants grown in CDM, suggesting that rather than one particular amino acid (i.e., proline) supporting growth on collagen, the acquisition of proline, arginine, and glutamate from collagen supports growth (Fig. 4B). In support of what we observed in the initial animal experiments (Fig. 1C), reduced growth was observed with the Δ*gudB* mutant, indicating that the peptides and/or free amino acids acquired from degraded collagen were not sufficient to support growth in the absence of glutamate or the amino acids that can be converted into glutamate. Overall, these data demonstrate that activated MMP-9 and Aur cleave collagen into peptides and free amino acids that can then be used to support S. aureus growth under nutrient-limited conditions. Additionally, particular amino acids were not required for growth, suggesting that the amount of collagen present in this assay provides abundant peptides and free amino acids to support growth even in media lacking particular essential amino acids.

### Peptide transport by Opp3 is important for growth on protease-degraded collagen.

**FIG 5:**
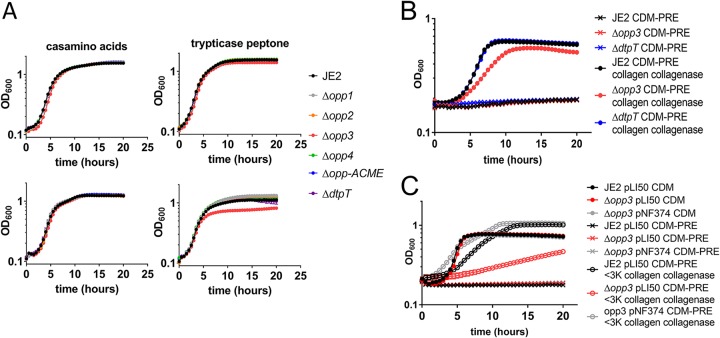
Opp3 transports peptides released from degraded collagen. (A) Growth curves of S. aureus JE2, Δ*opp1*, Δ*opp2*, Δ*opp3*, Δ*opp4*, Δ*opp-ACME*, and Δ*dtpT* strains in media supplemented with 1% Casamino Acids or 1% Trypticase peptone. Shown are growth in CDM (upper) and growth in CDM with limited (25%) amino acids (lower). (B) Growth curves of S. aureus JE2, Δ*opp3*, and Δ*dtpT* strains in CDM-PRE and CDM-PRE supplemented with collagenase-treated collagen. (C) Growth curves of S. aureus JE2 pLI50 (empty vector), Δ*opp3* pLI50, and Δ*opp3* pNF374 (pLI50::*opp3*) strains in CDM, CDM-PRE, or CDM-PRE supplemented with <3,000-MW (<3K) filtrate of collagen treated with collagenase. Briefly, collagen was incubated with collagenase and then separated by a size exclusion column (MWCO of 3,000), so only those peptides smaller than an MW of 3,000 were provided in the growth curve. Data are represented as means ± SEM (*n* = 3).

**FIG 6:**
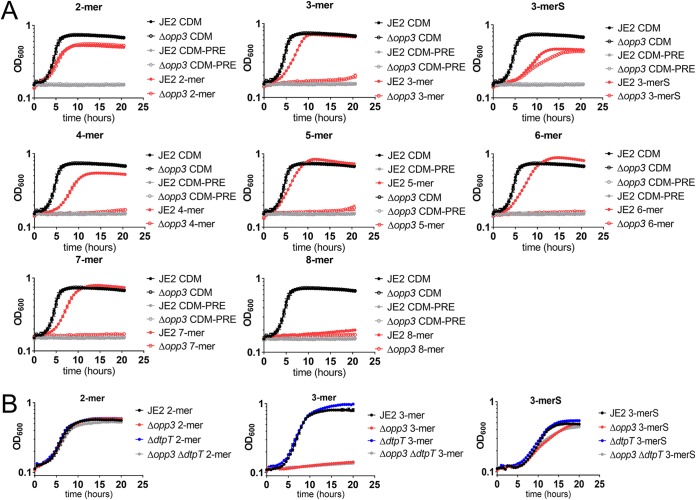
Two- to 7-mer peptides are transported by Opp3. (A) Growth curves of S. aureus JE2 and Δ*opp3* strains in CDM, CDM-PRE, and CDM-PRE supplemented with 13 μM the following peptides: 2-mer (PR), 3-mer (PRE), 3-merS (SPR), 4-mer (SPRE), 5-mer (SPRES), 6-mer (SSPRES), 7-mer (SSPRESS), and 8-mer (SSSPRESS). (B) Growth curves of S. aureus JE2, Δ*opp3*, Δ*dtpT*, and Δ*opp3* Δ*dtpT* strains in CDM and CDM-PRE supplemented with 13 μM the following peptides: 2-mer (PR), 3-mer (PRE), and 3-merS (SPR). Data are represented as means ± SEM (*n* = 3).

Collagen, once degraded by proteases, is sufficient for growth of S. aureus in media lacking proline, arginine, and glutamate (Fig. 4A). We sought to determine if S. aureus required specific oligopeptide transporters to import the collagen peptides to support growth. S. aureus JE2 encodes five complete putative oligopeptide transporters (49). To determine which of these annotated transporters are important for peptide transport, transposon mutants of the five oligopeptide transporters were grown in CDM, CDM supplemented with Casamino Acids, and CDM supplemented with Trypticase peptone. It should be noted that the medium supplemented with Casamino Acids was used as a peptide-free control that added supplemental nutrients into the media and that none of the oligopeptide transporter mutants had a growth defect under these conditions. In contrast, the Δ*opp3* mutant had a growth defect when grown in CDM supplemented with Trypticase peptone, suggesting that Opp3 is important for transport of peptides (Fig. 5A, upper). Furthermore, we reduced the amounts of amino acids present in the CDM by 4-fold and found that the defect was even more severe for the Δ*opp3* mutant (Fig. 5A, lower). This confirms previous studies, which also identified Opp3 as the primary oligopeptide transporter that supports growth on peptides (45). Additionally, the dipeptide transporter DtpT has also been shown to transport short peptides (2 to 3 amino acids) and support growth. A Δ*dtpT* mutant alone did not have a growth defect in media supplemented with peptides, suggesting that the contribution of DtpT to peptide transport required for growth is minimal (Fig. 5A). Opp3 has been shown to transport peptides that range in size from 3 to 8 amino acids (45). To determine if Opp3 is important for supporting growth on degraded collagen, collagen digested with collagenase was added to CDM and CDM-PRE. As the cell must transport either free amino acids or peptide fragments from digested collagen to sustain growth in CDM-PRE, it was not surprising that the Δ*opp3* mutant had a mild growth defect in CDM-PRE supplemented with digested collagen (Fig. 5B). Moreover, when we isolated peptides that were smaller than a molecular weight (MW) of 3,000 from the digested collagen and added the peptides with an MW of less than 3,000 to CDM-PRE, we observed further reduced growth in the Δ*opp3* mutant compared to that of the wild type (Fig. 5C). Furthermore, growth was restored to levels observed in the wild-type strain when *opp3* was expressed *in trans* in the Δ*opp3* mutant (Fig. 5C). These data suggest that small peptides released by collagen degradation are responsible for most of the growth observed in media lacking the essential amino acids arginine, proline, and glutamate. To determine the limits of the size of peptides that can support growth under our conditions, peptides ranging from 2 (proline-arginine) to 13 (serine_5_-proline-arginine-glutamate-serine_5_) amino acids, including proline, arginine, glutamate, and serine (see Table S2 for peptide composition), were synthesized. In CDM-PRE supplemented with the individual peptides, we observed growth of the wild-type strain when the 2-mer through the 7-mer were added, but peptides comprised of 8 to 13 amino acids failed to support robust growth (Fig. 6A and Fig. S3A). In the Δ*opp3* mutant, no growth occurred with peptides longer than two amino acids, suggesting that Opp3 is responsible for transport of 3- to 7-mer peptides. This phenotype was complementable with *opp3* expressed in *trans* in the Δ*opp3* mutant (Fig. S3B). It should be noted that we observed growth of the Δ*opp3* mutant when grown with the 3-mer encoding SPR (3-merS) but not PRE (3-mer), suggesting that an additional transporter can transport 2- and some 3-mers. To determine if DtpT is responsible for the transport of smaller 2- and 3-mers, Δ*dtpT* and Δ*dtpT* Δ*opp3* mutants were grown in CDM-PRE supplemented with the 2-mer (PR), 3-mer (PRE), and 3-merS (SPR). The *dtpT opp3* double mutant phenocopied the *opp3* mutant, suggesting that there are additional peptide transporters that can transport smaller 2- and 3-mers (Fig. 6B). Overall, these data suggest that Opp3 is important for nutrient acquisition from the environment.

10.1128/mBio.02553-18.3 FIG S3 (A) Growth curves of S. aureus JE2 and Δ*opp3* strains in CDM, CDM-PRE, and CDM-PRE supplemented with 13 μM the following peptides: 9-mer (SSSPRESSS), 10-mer (SSSSPRESSS), 11-mer (SSSSPRESSSS), 12-mer (SSSSSPRESSSS), and 13-mer (SSSSSPRESSSS). Data are represented as means ± SEM, *n* = 3. (B) Growth curves of S. aureus JE2 pLI50 (empty vector), Δ*opp3* pLI50, and Δ*opp3* pNF374 (pLI50::*opp3*) strains in CDM or CDM-PRE supplemented with 13 μM the 3-mer (PRE) or 8-mer (SSSPRESS). Data are represented as means ± SEM, *n* = 3. Download FIG S3, PDF file, 0.5 MB. Copyright © 2019 Lehman et al. 2019 Lehman et al. This content is distributed under the terms of the Creative Commons Attribution 4.0 International license.

10.1128/mBio.02553-18.6 TABLE S2 Synthesized peptides. Download Table S2, DOCX file, 0.01 MB. Copyright © 2019 Lehman et al. 2019 Lehman et al. This content is distributed under the terms of the Creative Commons Attribution 4.0 International license.

### Presence of peptides induces aureolysin expression.

**FIG 7:**
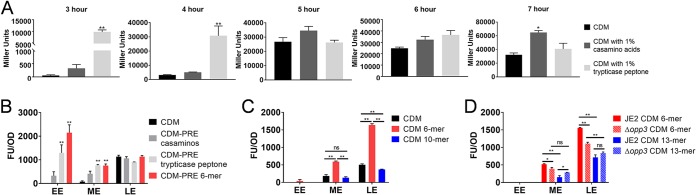
Opp3 acts as a sensor to induce aureolysin in response to the presence of peptides. (A) S. aureus JE2 pNF323 (P*_aur_*::*lacZ*) was grown in 30 ml CDM, CDM supplemented with 1% Casamino Acids, or CDM supplemented with 1% Trypticase peptone. Samples were taken every hour starting at early exponential phase (3 h) until late exponential phase (7 h), and β-galactosidase assays were performed. Miller units were calculated based on protein concentrations. S. aureus JE2 pCM13 (P*_aur_*::*gfp*) growth curves were performed in CDM-PRE (B) or CDM (C) supplemented with 1% Casamino Acids, 1% Trypticase peptone, or 13 μM the synthesized 6- or 10-mer (outlined in Table S2) in 96-well plates. (D) S. aureus JE2 pCM13 and Δ*opp3* pCM13 strain growth curves were performed in CDM supplemented with 13 μM the synthesized 6- or 13-mer (outlined in Table S2) in 96-well plates. Growth was observed at the OD_600_ and fluorescence at 490 nm/520 nm every 0.5 h. Fluorescent units (FU)/OD_600_ were determined for each time point, with data displayed as arbitrary time points represented at the growth phase, as the various peptides affected the growth rate of the bacteria. EE, early exponential; ME, mid-exponential; LE, late exponential. Data are represented as means ± SEM, *n* = 3 (A, B, and D) and *n* = 4 (C). Tukey's multiple-comparison test was performed. *P* values were <0.05 (*) and <0.05 (**) compared to CDM unless otherwise noted.

The ability of S. aureus to sense and respond to its changing environments allows it to colonize a variety of sites within the host. The metalloprotease aureolysin has the ability to cleave collagen as well as activate MMP-9, which can also cleave collagen (Fig. 3). Collagen peptides are sufficient to support S. aureus growth under nutrient-limited conditions (Fig. 4). Due to the ability of Aur to cleave collagen and support growth, we sought to determine if *aur* expression was induced by the presence of peptides. Therefore, a P*_aur_*::*lacZ* reporter plasmid was transduced into S. aureus JE2 and grown in CDM in the presence of additional Casamino Acids or Trypticase peptone. During early exponential phase, *aur* reporter expression was induced by the presence of peptides in Trypticase peptone, but additional Casamino Acids did not induce *aur* reporter expression, indicating that peptides, but not free amino acids, induce *aur* reporter expression (Fig. 7A). Moreover, when the P*_aur_*::*gfp* reporter plasmid was transduced into JE2, the presence of the synthesized 6-mer induced early *aur* reporter expression (Fig. 7B) in CDM-PRE, similar to what we observed in the P*_aur_*::*lacZ* reporter. Interestingly, only the peptides that fall within the range of Opp3 transport, those in the range of 3 to 7 amino acids, most strongly induced *aur* reporter expression (Fig. 7C). Also of note, it appears as though the induction is dependent on nutrient conditions, as induction by the 6-mer in CDM occurred later than what was observed in CDM-PRE (Fig. 7B). These data concur with previous observations by Borezee-Durant et al., in which decreased *sspB* and *aur* expression in an *opp3* mutant was observed (42). To test if Opp3 is required for differential expression of *aur* in the presence of peptides, the P*_aur_*::*gfp* plasmid was transduced into the Δ*opp3* mutant. When grown in the presence of the 6-mer, a peptide transported by Opp3, there is a slight reduction in P*_aur_*::*gfp* induction both at mid- and late exponential phase. In the presence of the 13-mer, which cannot be transported by Opp3, the Δ*opp3* mutation did not reduce *aur* expression, suggesting that Opp3 functions as a sensor for the presence of peptides.

Lastly, we sought to determine if Opp3 and aureolysin were required for proliferation within a skin abscess. C57BL/6 mice were inoculated with 1 × 10^6^ CFU Δ*hla*, Δ*hla Δopp3*, or Δ*hla Δaur* mutant. In all strains, we recovered similar numbers of bacteria, suggesting that these single mutations do not hinder the ability of the bacteria to survive within the skin abscess (Fig. S4). These results are not that surprising, as there are multiple proteases, including staphopain B and MMP-9, that can cleave collagen. Moreover, there are also additional host proteins available that may also serve as a nutrient reservoir for S. aureus. Additionally, the expression levels of Opp3 have not been characterized *in vivo*. Overall, these data suggest that the skin abscess milieu is complex, with a variety of host proteins that can support growth, with Opp3 and Aur having an important yet not essential role in nutrient acquisition.

10.1128/mBio.02553-18.4 FIG S4 Bacterial burdens of 7-week-old C57BL/6 mice subcutaneously infected with 1 × 10^6^ CFU of S. aureus Δ*hla*, Δ*hla* Δ*opp3*, or Δ*hla* Δ*aur* strain 5 days postinfection. No statistical significance was found between the strains as determined by Kruskal-Wallis test (*n* = 10). Data are representative of 2 independent studies. Download FIG S4, PDF file, 0.3 MB. Copyright © 2019 Lehman et al. 2019 Lehman et al. This content is distributed under the terms of the Creative Commons Attribution 4.0 International license.

## DISCUSSION

S. aureus can colonize a variety of niches within the host, suggesting that it has the ability to adapt its metabolism to support growth under diverse nutrient conditions. In support of this hypothesis, it has been observed that in the glucose-rich environment of the liver, the carbon catabolite repressor CcpA is important for proliferation, but the Δ*ccpA* mutant had little effect on bacterial burden in the kidney (61). Furthermore, mutants defective in aerobic respiration were unable to colonize the heart and liver but still caused infection in the kidney (62). In the kidney, anaerobic fermentation is important for virulence, with fermentation mutants having reduced abscess formation (63). Our data suggest that catabolism of peptides and free amino acids is important for proliferation within a skin abscess, with a Δ*pckA* gluconeogenesis mutant and Δ*gudB* glutamate dehydrogenase mutant having reduced bacterial burden and, importantly, resulting in bacterial burdens below the limit of detection in a number of animals (Fig. 1C). Moreover, we showed that collagen, a host protein abundant at the site of infection, can serve as a nutrient reservoir for S. aureus proliferation within the abscess. Collagen can be digested by the host protease MMP-9, which is also abundant in infected tissue (Fig. 2). In addition to MMP-9, we identified that the staphylococcal proteases aureolysin and staphopain B also have the ability to digest collagen (Fig. 3). We hypothesize that both staphylococcal and host proteases are important for liberation of peptides and free amino acids into the abscess milieu for uptake by S. aureus. Once the peptides are liberated, Opp3 is the primary oligopeptide transporter responsible for transport of the peptides into S. aureus to be catabolized to support growth (Fig. 5). Lastly, the presence of peptides induces aureolysin expression (Fig. 7), with Opp3 acting as a sensor, suggesting that S. aureus has a mechanism for sensing and relaying the message that peptides are present in the environment and inducing expression of proteases that aid in the digestion of peptides. Overall, these data outline one pathway by which S. aureus acquires nutrients from host-derived sources.

Defining the nutrients that are available at the various niches in which S. aureus proliferates *in vivo* is difficult. The milieu surrounding the bacteria during an infection is ever changing, and the tools available for *in vivo* detection of nutrients are limited. Despite limited tools, nutrients available in a skin abscess have begun to be elucidated. Glucose is required for the initial establishment of an infection (12), and oxygen levels are presumably depleted within the abscess (12). Additionally, recent studies have determined that the skin abscess becomes glucose depleted as the infection progresses (20). Further confounding our ability to determine the nutrients available in a skin abscess is the differential disease outcomes that occur during a skin and soft-tissue infection. We have noted, as have others, that during an S. aureus skin and soft-tissue infection, some infections result in abscess formation, with little to no visible lesion in the epidermis (see Fig. S1A in the supplemental material). Other infections result in dermonecrosis, as defined by the necrosis of the dermis and epidermis. On visual examination, the dermonecrosis has a large, usually flat, lesion (Fig. S1B). These infections result in two very different environments in which S. aureus must proliferate. In these studies, we sought to control for the disease outcome of skin and soft-tissue infections to define what is specifically required metabolically while S. aureus is within a skin abscess. The formation of a dermonecrotic lesion is highly dependent on the initial inoculum of bacteria injected subcutaneously. In our experience, any inoculum higher than 5 × 10^6^ CFU results primarily in dermonecrosis in the C67BL/6 mouse background. Even with lower initial inocula, some animals still develop dermonecrotic lesions associated with the abscess. To address this phenomenon, we sought to genetically alter S. aureus to select for abscess formation rather than dermonecrosis. It is well characterized throughout the literature that alpha toxin, encoded by *hla*, is the primary virulence factor responsible for dermonecrosis (7–10). In the Δ*hla* background, we observed abscess formation with no dermonecrosis associated with the infection (Fig. 1A). Therefore, we were able to probe whether gluconeogenesis was important within a skin abscess environment without the confounding effects of dermonecrosis. Importantly, we observed that the Δ*pckA* and Δ*gudB* mutants, neither of which can grow when amino acids are the sole carbon source (21), had reduced bacterial burden in a skin abscess, suggesting that amino acids are a key carbon source in this environment. In contrast, other investigators have found that a mutation in *pckA* does not affect bacterial burden in the kidney and liver (12). In support of these previously published results, unpublished data from our laboratory suggest that the Δ*gudB* mutant also does not alter bacterial burden in the liver, kidney, and spleen in a bacteremia model, suggesting that amino acid catabolism is not essential at these sites of infection. Overall, these data highlight the nutritional differences and therefore differential metabolic requirements of S. aureus at specific sites of infection.

Collagen is one of the most abundant proteins available within the host (64), and it is clearly prominent within and surrounding the skin abscess (Fig. 2A). Other host proteins, including fibrinogen and serum proteins, such as albumin and hemoglobin, also may have a similar role in acting as a nutrient reservoir. For example, Bacillus anthracis utilizes hemoglobin, fibrinogen, and serum albumin as an amino acid source when grown in media mimicking serum (7). The ability to utilize hemoglobin as a nutrient reservoir was independent of the iron state of the protein and required the secreted protease lnhA1. Similarly, we observe that S. aureus can utilize collagen as a reservoir for essential amino acids (Fig. 4A). Importantly, we observed that in the presence of exogenous as well as endogenous protease production, collagen could support growth. There are other abundant host proteins available within the skin abscess, including fibrinogen, that also may serve as reservoirs for essential nutrients. Collagen in these studies was used as just one example of an abundant host protein that could be utilized by S. aureus as a nutrient reservoir. The degradation of proteins by MMP-9, aureolysin, and staphopain B should be tested on other host proteins to determine the specificity of the mechanisms described.

Collagen is a proline-rich protein, with proline comprising roughly 22% of the amino acids in the protein (64). In pancreatic ductal adenocarcinoma, proline derived from collagen promotes cell survival under nutrient-limited conditions, with MMPs aiding in the liberation of peptides and amino acids from the collagen (65). Importantly, proline metabolism has been identified as a key target in cancer treatment, as proline metabolism can support cellular proliferation, facilitate metastasis to distant organs, and prevent apoptosis (66). Similarly, we hypothesized that collagen is targeted by S. aureus to acquire proline due to its key role in amino acid metabolism. Based on previous studies in which proline was found to be the precursor for arginine biosynthesis (5) and that proline was the primary amino acid utilized as a carbon source (21), we sought to determine if proline catabolism was essential for recovery of growth in CDM-PRE supplemented with degraded collagen. We found that a *putA* mutant, which is unable to catabolize proline, was still able to grow in the presence of degraded collagen (Fig. 4B), suggesting that it is not essential to catabolize proline, even when collagen is the primary source of peptides. These data suggest that the abundance of additional amino acids present in collagen is able to support growth, even when proline is not able to be catabolized.

MMP-9 has a major role in tissue degradation and remodeling around the site of an infection. The activation of MMP-9 in the tissue is not fully understood, although it has been shown that trypsin, plasmin, human neutrophil elastase, as well as other MMPs, including MMP-2, MMP-3, and MMP-26, can activate MMP-9 (67). In our studies, we found that in addition to its ability to cleave collagen, aureolysin could also cleave, and therefore activate, MMP-9 (Fig. 3C and D). Other bacterial proteases have been found to have this ability to activate host proteases, suggesting exogenous proteins produced by the bacteria are altering host proteases to manipulate the site of the infection. Of the bacterial proteases that have been described to activate MMPs (e.g., Pseudomonas aeruginosa elastase [55, 58], Vibrio cholerae proteinase [55], thermolysin [55], and Enterococcus faecalis GelE [57]), many are metalloproteases. Aureolysin is also a metalloprotease (68). These observations suggest that the relationship between bacterial proteases and host proteases warrants further investigation.

The induction of *aur* transcription by peptides (Fig. 7) indicates that S. aureus has a mechanism for sensing the presence of peptides and altering gene expression in response. More specifically, S. aureus increases the transcription of a protease that has the ability to cleave proteins into usable peptide fragments that support proliferation. Additionally, the abundance of free amino acids alters protease production (42), suggesting that protease production is tightly linked to amino acid metabolism. Opp systems have been linked to virulence factor regulation in numerous bacterial species, although the signal transduction pathway has not been determined (69). There are a variety of mechanisms by which bacteria can sense nutrients in the environment (70). Initial results from our studies indicated that Opp3 is important for nutrient sensing, as the induction was more robust by peptides that can be transported by Opp3 (Fig. 7B and C). Moreover, there was previous data that showed that the level of *aur* transcript was decreased in an Δ*opp3* mutant (42). Indeed, we did observe reduced *aur* induction in the Δ*opp3* mutant when grown in the presence of peptides that can be transported by Opp3 (Fig. 7D). These data suggest that Opp3-dependent peptide transport alters protease production. Additional studies are focused on the mechanism by which Opp3 peptide transport is relayed to alter gene expression.

The ability of S. aureus to colonize a variety of niches in the host suggests an adaptive metabolism that allows for proliferation in the presence of various nutrients. Our studies define the skin abscess as a glucose-depleted environment that requires the bacteria to acquire peptides and free amino acids from the host for proliferation. Overall, these studies begin to elucidate the intricate mechanisms by which S. aureus is able sense the nutrients available at a site of infection and utilize host proteins as a nutrient reservoir to allow for proliferation and survival.

## MATERIALS AND METHODS

### Strains, plasmids, and growth conditions.

All of the strains and plasmids used in these studies are listed in Table S3 in the supplemental material. Defined *bursa aurealis* transposon mutants were obtained from the Nebraska Transposon Mutant Library and backcrossed to JE2 using Φ11 (71). Overnight cultures of bacteria were grown at 37°C with shaking at 250 rpm in TSB in the presence of erythromycin (5 μg ml^−1^) or chloramphenicol (10 μg ml^−1^) as needed. Growth curves were performed in CDM with no glucose (72) in 96-well plates in the TECAN device at 37°C with shaking at 250 rpm. The P*_aur_*::*lacZ* plasmid, pNF323, was created by amplifying the promoter region of aureolysin from JE2 chromosomal DNA with primers 3049 and 3050 (Table S4) and cloned into the BamHI and XhoI sites in pJB185. The *opp3BCDFA* complement plasmid, pNF374, was created by amplifying the entire *opp3BCDFA* operon, including its native promoter, with primers opp3 comp2 F and opp3 comp R (Table S4) and cloned into the KpnI and BamHI sites in pLI50.

10.1128/mBio.02553-18.7 TABLE S3 Bacterial strains and plasmids used in the study. Download Table S3, DOCX file, 0.02 MB. Copyright © 2019 Lehman et al. 2019 Lehman et al. This content is distributed under the terms of the Creative Commons Attribution 4.0 International license.

10.1128/mBio.02553-18.8 TABLE S4 Primers used in the study. Download Table S4, DOCX file, 0.01 MB. Copyright © 2019 Lehman et al. 2019 Lehman et al. This content is distributed under the terms of the Creative Commons Attribution 4.0 International license.

### Fluorescent collagen assays.

DQ collagen type I (ThermoFisher, Waltham, MA) assays were performed as directed by the user manual. Briefly, DQ collagen was diluted to a final concentration of 12.5 μg ml^−1^ in DQ collagen buffer (50 mM Tris-HCl, 150 mM NaCl, 5 mM CaCl_2_, pH 7.6). MMP-9 (BioLegend, San Diego, CA) was diluted to a concentration of 1 μg ml^−1^ in DQ collagen buffer and added at a final concentration of 0.05 μg ml^−1^. Aureolysin (Preparatis, Krakow, Poland) was diluted to a concentration of 1 μg ml^−1^ and added at a final concentration of 0.05 μg ml^−1^. When culture supernatant was added, cultures were grown overnight for approximately 16 h. Cells were spun down, and the supernatant was filtered through a 0.22-μm filter, with 15 μl of supernatant added to the 200-μl reaction mix. Plates were immediately read on a TECAN InfinitePro (Männedorf, Switzerland) at 480 nm/520 nm every 30 s.

### Zymogram.

Purified MMP-9 (1 μg ml^−1^ in DQ collagen buffer) was incubated with the following: APMA (Sigma, St. Louis, MO), purified aureolysin (1 μg ml^−1^ in DQ collagen buffer), JE2 supernatant (18 h of culture), or *sarA*::*tetM* supernatant (18 h of culture). The enzyme and supernatant were added at a 2:3 ratio (enzyme to supernatant). After 30 min of incubation at 37°C, 5 μl of sample was mixed with 3× SDS buffer (4% SDS, 20% glycerol, 0.125 M Tris-HCl, pH 6.8, 0.01% bromophenol blue) and loaded onto a 10% SDS-PAGE gel with 0.01% gelatin. The gel was run for approximately 1.5 h at 140 V. The gel was incubated for 1 h at room temperature in renaturation buffer (50 mM Tris-HCl buffer, pH 7.4, containing 5 mM CaCl_2_, 1 μM ZnCl_2_, and 2.5% Triton X-100) and then overnight at 37°C in development buffer (50 mM Tris-HCl buffer, pH 7.4, containing 5 mM CaCl_2_ and 1 μM ZnCl_2_). The gel was then stained with Coomassie to visualize zones of clearing.

### Collagen growth curves.

One hundred twenty μg of type I collagen (Sigma, St. Louis, MO) was incubated with 1 μg human recombinant MMP-9 and/or aureolysin in DQ buffer for 2 h at 37°C. As a control, 120 μg of type I collagen was incubated with 1 U of collagenase (Sigma, St. Louis, MO) or DQ buffer. The collagen solutions were added to CDM-PRE inoculated with S. aureus strains at a starting optical density at 600 nm (OD_600_) of 0.05. Optical density at 600 nm was observed every 30 min using the TECAN plate reader.

### Animal studies.

Seven-week-old C57B/6 mice (Charles River Laboratories, Wilmington, MA) were inoculated with 1 × 10^6^ CFU S. aureus subcutaneously. Prior to inoculation, the flanks of the mice were shaved and sterilized with 70% alcohol. Mice were sacrificed at 5 days postinoculation, and abscess and periabscess tissue was removed and either placed in O.C.T. (Fisher Scientific, Hampton, NH) and frozen for histological analysis or placed in phosphate-buffered saline (PBS) to be homogenized and analyzed for CFU counts. Histological samples were processed in the UNMC Core Tissue Facility. This study was conducted in accordance with the recommendations in the *Guide for the Care and Use of Laboratory Animals* of the National Institutes of Health (73). The animal protocol was approved by the Institutional Animal Care and Use Committee of the University of Nebraska Medical Center (protocol 11-049-06-FC).

### Immunohistochemistry.

Tissues from the skin abscess or surrounding sterile field were processed for immunofluorescence staining using primary antibodies for matrix metallopeptidase 9 (monoclonal mouse anti-MMP9; Abcam) and collagen type I (polyclonal rabbit anti-ColI; Millipore, Burlington, MA). Frozen tissue sections were fixed (100% methanol), blocked in 5% donkey serum, and incubated with primary antibodies (Arg-1, 1:100; iNOS, 1:100; MMP-9, 1:500; ColI, 1:40) diluted in 2% donkey serum overnight at 4°C. Sections were then incubated with donkey anti-mouse Alexa Fluor 488 and donkey anti-rabbit Alexa Fluor 648 (1:250; Jackson ImmunoResearch Laboratories, West Grove, PA) secondary antibodies and Hoechst stain (1:100; Molecular Probes, Eugene, OR) for 2 h at room temperature. The samples were washed and dried, and the glass slides were mounted with Slow Fade (Life Technologies, Carlsbad, CA). Confocal imaging was performed using a Zeiss 710 META laser scanning microscope (Carl Zeiss, Oberkochen, Germany).

### Western blotting and zymogram of skin abscess tissue.

Tissue homogenate was prepared from 7-week-old C57B/6 mice inoculated with 1 × 10^6^ CFU S. aureus subcutaneously. Control tissue was isolated from the same animal. Protein concentration of homogenates was determined using a Bio-Rad protein assay (Hercules, CA). Two μg of protein was loaded onto a 10% SDS-PAGE gel or a 10% SDS-PAGE gel with 0.01% gelatin. MMP-9 was detected with monoclonal mouse anti-MMP9 (Abcam, Cambridge, United Kingdom). The gelatin gel was incubated for 1 h at room temperature in renaturation buffer (50 mM Tris-HCl buffer, pH 7.4, containing 5 mM CaCl_2_, 1 μM ZnCl_2_, and 2.5% Triton X-100) and then overnight at 37°C in development buffer (50 mM Tris-HCl buffer, pH 7.4, containing 5 mM CaCl_2_ and 1 μM ZnCl_2_). The gel was then stained with Coomassie to visualize zones of clearing.

### Peptide transporter growth curves.

S. aureus strains were inoculated at an OD_600_ of 0.05 in CDM supplemented with 1% Casamino Acids (Becton Dickinson, Franklin Lakes, NJ) or 1% Trypticase peptone (Becton Dickenson, Franklin Lakes, NJ). Amino acids in the CDM were reduced by 4-fold (25% amino acid CDM) and supplemented with 1% Trypticase peptone or Casamino Acids. Collagen treated with 1 U collagenase for 45 min was spun down in a 3,000-molecular-weight-cutoff (MWCO) PES spin column (Sartorius AG, Göttingen, Germany). Filtrate was added to CDM-PRE inoculated to a final OD of 0.05. Synthetic peptides (Genemed Synthesis, San Antonio, TX) (Table S2) were added to CDM-PRE at a final concentration of 13 μM. Growth curves were performed in 96-well plates in the TECAN device at 37°C with shaking at 250 rpm.

### P*_aur_*::*lacZ* growth curves.

Overnight cultures of JE2 pNF323 were washed in PBS and diluted to a starting OD_600_ of 0.05 in 30 ml CDM, CDM with 1% Casamino Acids, and CDM with 1% Trypticase peptone in a 250-ml flask. The strains were grown at 37°C with shaking at 250 rpm, and samples were taken every hour during exponential phase. β-Galactosidase activity was assessed as previously described (74).

### Green fluorescent protein growth assays.

The JE2 pCM13 or *Δopp3* pCM13 strain was grown in 200 μl CDM or CDM-PRE supplemented with 0.5% Casamino Acids, 0.5% Trypticase peptone, or 13 mM synthetic peptone. Growth and fluorescence (490 nm/520 nm) were assessed in 96-well clear-bottomed black plates in the TECAN device at 37°C with shaking at 250 rpm.
