# Supplementary material for: Protease-Mediated Growth of Staphylococcus aureus on Host Proteins Is opp3 Dependent
Source: mBio. 2019 Apr 30;10(2):e02553-18. doi: 10.1128/mBio.02553-18 (PMC6495380; doi:10.1128/mBio.02553-18)
Supplement: FIG S1 [file mBio.02553-18-sf001.pdf]

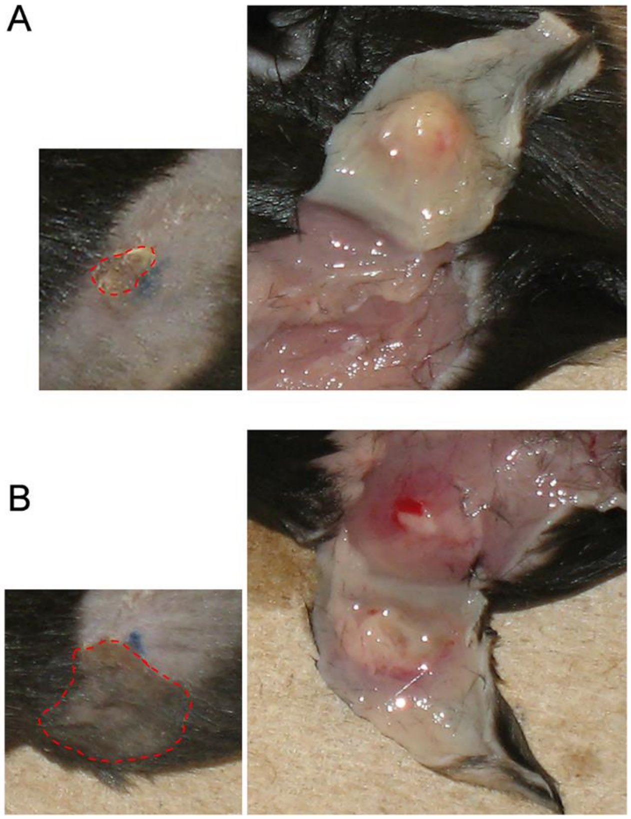

Figure S1. Images of 7-week old C57BL/6 mice infected with 1 x 10^6 CFU *S. aureus* JE2 resulting in **A) skin** abscess formation or **B)** dermonecrotic lesion. Left panel is image of mouse prior to abscess or necrotic lesion excision. Right panel is image of the underside of the abscess or necrotic lesion prior to excision. The red dashed line outlines each lesion.
