# Supplementary material for: Protease-Mediated Growth of Staphylococcus aureus on Host Proteins Is opp3 Dependent
Source: mBio. 2019 Apr 30;10(2):e02553-18. doi: 10.1128/mBio.02553-18 (PMC6495380; doi:10.1128/mBio.02553-18)
Supplement: FIG S2 [file mBio.02553-18-sf002.pdf]

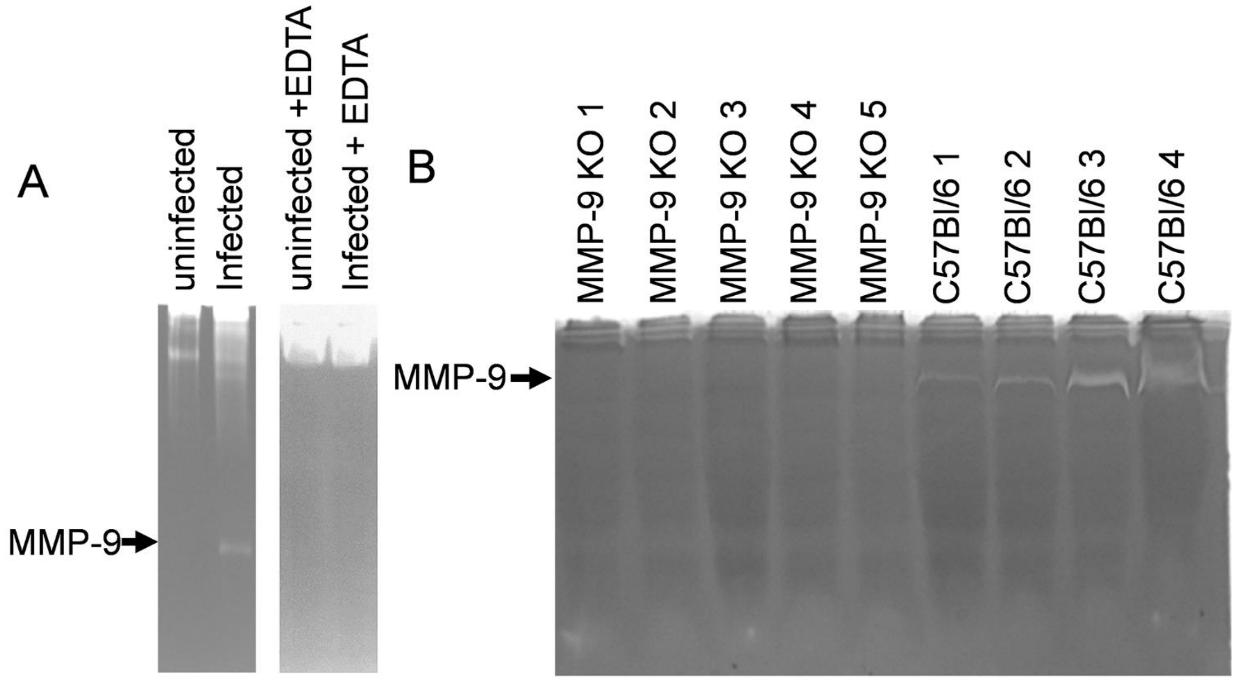

Figure S2. A) Supernatant from uninfected kidney and *S. aureus* JE2-infected kidney homogenate was run on a 10% SDS-page gel with 0.5 mg ml^{-1} type I collagen added. In the left panel, a 100 kDa collagenase (black arrow) was present in the infected kidney homogenate lane but absent in the uninfected kidney homogenate lane. The collagenase activity was abolished when EDTA was added. **B)** Collagen zymogram of *S. aureus* JE2-infected kidney homogenate from MMP-9 knockout and C57BL/6 mice.
