# Supplementary material for: Protease-Mediated Growth of Staphylococcus aureus on Host Proteins Is opp3 Dependent
Source: mBio. 2019 Apr 30;10(2):e02553-18. doi: 10.1128/mBio.02553-18 (PMC6495380; doi:10.1128/mBio.02553-18)
Supplement: TABLE S1 [file mBio.02553-18-st001.docx]

**Table S1: Rates of collagen degradation**

|  | Rate FU/min ± SE |
| --- | --- |
| MMP-9 | 0.08333 ± 0.09279 |
| Δ*sarA* Δ*aur* | 1.162 ± 0.02095 |
| Δ*sarA* Δ*aur* MMP-9 | 1.285 ± 0.01833 |
| APMA MMP-9 | 1.368 ± 0.1168 |
| Δ*sarA* Δ*scpA* Δ*sspB* | 2.039 ± 0.02763 |
| Δ*sarA* Δ*sspB* | 2.082 ± 0.02765 |
| Δ*sarA* Δ*sspB* MMP-9 | 2.548 ± 0.04174 |
| Δ*sarA* Δ*scpA* Δ*sspB* MMP-9 | 2.705 ± 0.03217 |
| Δ*sarA* | 4.724 ± 0.02777 |
| Δ*sarA* Δ*scpA* | 5.55 ± 0.05779 |
| Δ*sarA* Δ*scpA* MMP-9 | 6.556 ± 0.05557 |
| Δ*sarA* MMP-9 | 7.47 ± 0.09161 |
