# Supplementary material for: Protease-Mediated Growth of Staphylococcus aureus on Host Proteins Is opp3 Dependent
Source: mBio. 2019 Apr 30;10(2):e02553-18. doi: 10.1128/mBio.02553-18 (PMC6495380; doi:10.1128/mBio.02553-18)
Supplement: FIG S3 [file mBio.02553-18-sf003.pdf]

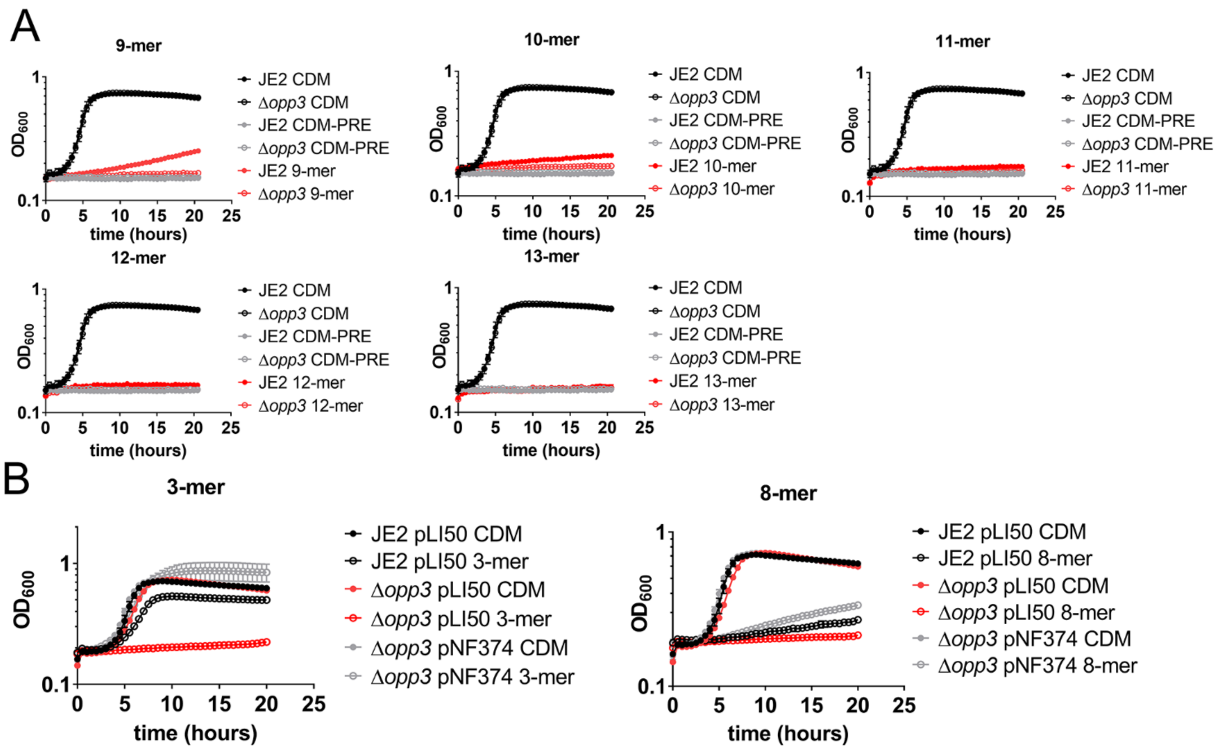

Figure S3. A) Growth curves of *S. aureus* JE2 and Δ*opp3* in CDM, CDM-PRE, and CDM-PRE supplemented with 13 μM of following peptides: 9-mer (SSSPRESSS), 10-mer (SSSSPRESSS), 11-mer (SSSSPRESSSS), 12-mer (SSSSSPRESSSS), 13-mer (SSSSSPRESSSS). Data are represented by the mean ± SEM, n=3. **B)** Growth curves of *S. aureus* JE2 pLI50 (empty vector), Δ*opp3* pLI50, and Δ*opp3* pNF374 (pLI50::*opp3*) in CDM or CDM-PRE supplemented with 13 μM of the 3-mer (PRE) or 8-mer (SSSPRESS). Data are represented by the mean ± SEM, n=3.
