# Supplementary material for: Protease-Mediated Growth of Staphylococcus aureus on Host Proteins Is opp3 Dependent
Source: mBio. 2019 Apr 30;10(2):e02553-18. doi: 10.1128/mBio.02553-18 (PMC6495380; doi:10.1128/mBio.02553-18)
Supplement: TABLE S2 [file mBio.02553-18-st002.docx]

**Table S2: Synthesized peptides**

| name | amino acid sequence |
| --- | --- |
| 2-mer | PR |
| 3-mer | PRE |
| 3-merS | SPR |
| 4-mer | SPRE |
| 5-mer | SPRES |
| 6-mer | SSPRES |
| 7-mer | SSPRESS |
| 8-mer | SSSPRESS |
| 9-mer | SSSPRESSS |
| 10-mer | SSSSPRESSS |
| 11-mer | SSSSPRESSSS |
| 12-mer | SSSSSPRESSSS |
| 13-mer | SSSSSPRESSSSS |
