# Supplementary material for: Protease-Mediated Growth of Staphylococcus aureus on Host Proteins Is opp3 Dependent
Source: mBio. 2019 Apr 30;10(2):e02553-18. doi: 10.1128/mBio.02553-18 (PMC6495380; doi:10.1128/mBio.02553-18)
Supplement: FIG S4 [file mBio.02553-18-sf004.pdf]

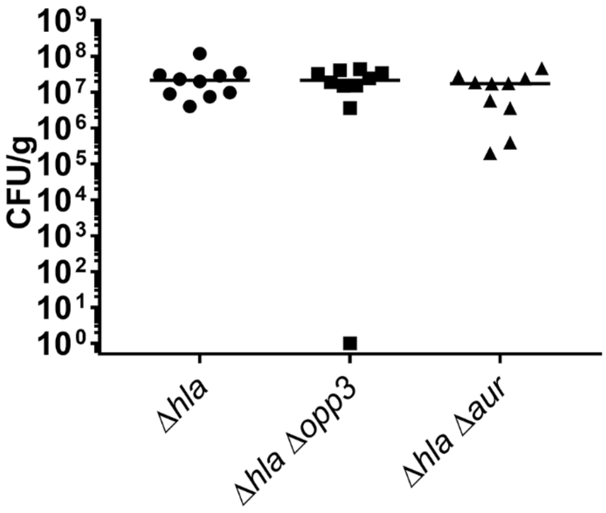

Figure S4. Bacterial burdens of 7-week old C57BL/6 mice subcutaneously infected with 1 x 10^6 CFU of *S. aureus* Δ*hla*, Δ*hla* Δ*opp3*, or Δ*hla* Δ*aur* 5 days post infection. No statistical significance between the strains as determined by Kruskal-Wallis Test, n=10. Data are representative of 2 independent studies.
