# Supplementary material for: Protease-Mediated Growth of Staphylococcus aureus on Host Proteins Is opp3 Dependent
Source: mBio. 2019 Apr 30;10(2):e02553-18. doi: 10.1128/mBio.02553-18 (PMC6495380; doi:10.1128/mBio.02553-18)
Supplement: TABLE S3 [file mBio.02553-18-st003.docx]

**Table S3. Bacterial strains and plasmids used in study**

| **Bacterial Strain or plasmid** | **Relevant phenotype** | **source** |
| --- | --- | --- |
| pCM13 | P*_aur_::gfp;* erm^R^ | (1) |
| pJB185 | Promoterless codon-optimized *lacZ*; Amp^r^ Cm^r^ | (2) |
| pNF323 | P*_aur_::lacZ;* cm^R^ | This study |
| pLI50 | *E. coli*- *S. aureus* shuttle vector; Amp^r^ Cm^r^ | (3) |
| pNF374 | pLI50::*opp3BCDFA* (under the control of its native promoter) | This study |
| *S. aureus* JE2 | Wildtype USA300 isolate | (4) |
| *S. aureus hla::tet* (Δ*hla)* | *hla::ΦΝΕ* with erm cassette replaced with *tet* | This study |
| *S. aureus hla::tet opp3B::ΦΝΕ* (Δ*hla* Δ*opp3)* | *opp3B::ΦΝΕ* backcrossed into *hla::tet* using Φ11 | This study |
| *S. aureus hla::tet aur::ΦΝΕ*  (Δ*hla* Δ*aur*) | *aur::ΦΝΕ* backcrossed into *hla::tet* using Φ11 | This study |
| *S. aureus hla::tet pckA::ΦΝΕ* (Δ*hla* Δ*pckA)* | *hla::tet* backcrossed into *pckA::ΦΝΕ* using Φ11 | This study |
| *S. aureus hla::tet gudB::ΦΝΕ*  (Δ*hla* Δ*gudB)* | *hla::tet* backcrossed into *gudB::ΦΝΕ* using Φ11 | This study |
| *S. aureus opp3B::tet* (Δ*opp3)* | *opp3B::ΦΝΕ* (SAUSA300_0887) with *erm* cassette replaced with *tet* | This study |
| *S. aureus opp1A::ΦΝΕ* (Δ*opp1)* | *opp1A::ΦΝΕ* (SAUSA300_2411) backcrossed into JE2 using Φ11 | This study |
| *S. aureus opp2B::ΦΝΕ* (Δ*opp2)* | *opp2B::ΦΝΕ* (SAUSA300_1276) backcrossed into JE2 using Φ11 | This study |
| *S. aureus opp4A::ΦΝΕ* (Δ*opp4)* | *opp4A::ΦΝΕ* (SAUSA300_0892) backcrossed into JE2 using Φ11 | This study |
| *S. aureus oppA-ACME::ΦΝΕ* (Δ*opp-ACME)* | *oppA-ACME::ΦΝΕ* (SAUSA300_0073) backcrossed into JE2 using Φ11 | This study |
| *S. aureus dtpT::ΦΝΕ* (Δ*dtpT)* | *dtpT::ΦΝΕ* backcrossed into JE2 using Φ11 (SAUSA300_0712) | This study |
| *S. aureus dtpT::ΦΝΕ opp3::tet* (Δ*opp3* Δ*dtpT*) | *opp3::tet* backcrossed into *dtpT::ΦΝΕ* using Φ11 | This study |
| *S. aureus gudB::ΦΝΕ* (Δ*gudB)* | *gudB::ΦΝΕ* backcrossed into JE2 using Φ11 | (5) |
| *S. aureus rocA::ΦΝΕ* (Δ*rocA)* | *rocA::ΦΝΕ* backcrossed into JE2 using Φ11 | (5) |
| *S. aureus rocD::ΦΝΕ* (Δ*rocD)* | *rocD::ΦΝΕ* backcrossed into JE2 using Φ11 | (5) |
| *S. aureus putA::ΦΝΕ* (Δ*putA)* | *putA::ΦΝΕ* backcrossed into JE2 using Φ11 | (5) |

1. Mootz JM, Malone CL, Shaw LN, Horswill AR. 2013. Staphopains modulate *Staphylococcus aureus* biofilm integrity. Infect Immun 81:3227-38.

2. Krute CN, Rice KC, Bose JL. 2017. VfrB Is a Key Activator of the *Staphylococcus aureus* SaeRS Two-Component System. J Bacteriol 199.

3. Lee CY, Buranen SL, Ye ZH. 1991. Construction of single-copy integration vectors for *Staphylococcus aureus*. Gene 103:101-5.

4. Fey PD, Endres JL, Yajjala VK, Widhelm TJ, Boissy RJ, Bose JL, Bayles KW. 2013. A genetic resource for rapid and comprehensive phenotype screening of nonessential *Staphylococcus aureus* genes. mBio 4:e00537-12.

5. Halsey CR, Lei S, Wax JK, Lehman MK, Nuxoll AS, Steinke L, Sadykov M, Powers R, Fey PD. 2017. Amino Acid Catabolism in *Staphylococcus aureus* and the Function of Carbon Catabolite Repression. mBio 8.
