# Supplementary material for: Protease-Mediated Growth of Staphylococcus aureus on Host Proteins Is opp3 Dependent
Source: mBio. 2019 Apr 30;10(2):e02553-18. doi: 10.1128/mBio.02553-18 (PMC6495380; doi:10.1128/mBio.02553-18)
Supplement: TABLE S4 [file mBio.02553-18-st004.docx]

**Table S4: Primers used in study**

| **Name** | **Sequence (5’ to 3’)** | **Target** |
| --- | --- | --- |
| 3049 | CC*GGATCC*AATAATGGCATATTAAGGCTAGAG | P*_aur_*-F |
| 3050 | CC*GTCGAC*CTTAAAAACAGCTTATAAATAAAATATTAATT | P*_aur_*-R |
| opp3 comp2 F | CC*GGTACC*AAATATGTAATGATAGTATGGC | *opp3BCDFA* |
| opp3 comp R | CC*GGATCC*TTATTTTTTCTTCTTACCTGTTTC | *opp3BCDFA* |

|  |  |
| --- | --- |

*Restriction site denoted in italics
